# Analyzing the International Exergy Flow Network of Ferrous Metal Ores

**DOI:** 10.1371/journal.pone.0106617

**Published:** 2014-09-04

**Authors:** Hai Qi, Haizhong An, Xiaoqing Hao, Weiqiong Zhong, Yanbing Zhang

**Affiliations:** 1 School of Humanities and Economic Management, China University of Geosciences, Beijing, China; 2 Key Laboratory of Carrying Capacity Assessment for Resource and Environment, Ministry of Land and Resources, Beijing, China; 3 Department of Teaching Affairs, Hebei University of Science and Technology, Shijiazhuang, China; 4 Lab of Resources and Environmental Management, China University of Geosciences, Beijing, China; North China Electric Power University, China

## Abstract

This paper employs an un-weighted and weighted exergy network to study the properties of ferrous metal ores in countries worldwide and their evolution from 2002 to 2012. We find that there are few countries controlling most of the ferrous metal ore exports in terms of exergy and that the entire exergy flow network is becoming more heterogeneous though the addition of new nodes. The increasing of the average clustering coefficient indicates that the formation of an international exergy flow system and regional integration is improving. When we contrast the average out strength of exergy and the average out strength of currency, we find both similarities and differences. Prices are affected largely by human factors; thus, the growth rate of the average out strength of currency has fluctuated acutely in the eleven years from 2002 to 2012. Exergy is defined as the maximum work that can be extracted from a system and can reflect the true cost in the world, and this parameter fluctuates much less. Performing an analysis based on the two aspects of exergy and currency, we find that the network is becoming uneven.

## Introduction

Ferrous metals (iron, chromium and manganese) are the main raw materials for the steel industry and play important roles in the national economy. Ferrous metal production accounts for approximately 95% of the world's metal production [1], [2]. Ferrous metal ores are processed to produce ferrous metals; however, their distribution in the world is extremely unbalanced. Under the drivers of demand and supply, many countries meet their needs by importing and exporting ferrous metal ores. Trade networks of ferrous metal ores are thus formed and are the subject of numerous studies [3]. Some researchers analyze the world trade network based on currency [4], [5] or based on the quantity of the commodity [6] to explore the pattern of the international trade network and provide a reference for trade policy.

Traditionally, people value the combination of socio-economic material in terms of economic currency. However, the monetary valuations lack the type of scientific definition that is based on energetic or physical explanations.

Unlike monetary measurements, some scholars who study ecological economics prefer to consider all processes and activities in terms of their energetics. Using this approach, it is possible to apply the laws of thermodynamics to social and ecological problems. In particular, the concept of exergy provides a unified indicator of different forms of material and energy flows based on evaluating the distance from the studied system to thermodynamic equilibrium [7], [8]. That is, exergy is defined as the maximum work that can be extracted from a system when this system moves toward thermodynamic equilibrium with a reference state. Exergy can be thought of as a measure of the quality or potential of a system to cause change. In contrast to energy, exergy is not subject to the conservation law except for ideal or reversible processes and, instead, is consumed or destroyed due to the unavoidable irreversibility within any real process. Unlike energy flow, which only concerns quantity, exergy is a measure of the quantity and quality of energy resources [7], [8].

In the 1950s, the exergy analysis method was used in thermodynamic engineering process evaluation and thermo-chemical system analysis. This method is capable of assessing work based on the Second Law of Thermodynamics. Thus, this method became an accepted method in thermal processing analysis [9], [10]. Subsequently, exergy analysis was developed into a measure of ecological complexity regarding how far an observed ecosystem is from a reference environment [11] and was used to determine the natural resource availability and environmental effect [12]–[14]. Exergy analysis provides a useful method with physical meaning to evaluate resource degradation.

For exergy to be able to unify the material, energy, and information, Wall [7], [8] creatively introduced exergy into the accounting work of social resource consumption. Recently, many researchers have applied exergy analysis for energy-utilization assessments to attain energy-saving strategies [10]. Some scholars have studied different national and sectorial levels applying exergy evaluation and have achieved results in these case studies: (1) For national levels, Japan [15], Sweden [8], Norway [16], the United States [17], China [18], the UK [19], [20], Italy [21] and some other countries have applied exergy evaluations. These studies analyzed the energy and material flow and efficiency from the perspective of exergy and assisted the country's policy makers in making energy and resource decisions. (2) In the social sectorial level, Dincer and his group published a series of papers on the transportation industry and domestic, public and private sectors in Saudi Arabia [22]–[27], and Chen et al. studied China [28] to assess the “resource content” of social input as well as environmental emissions [29], [30]. Chen and his group studied the types of resource and different industries of Chinese society from the exergy perspective and proposed some suggestions to policymakers to improve the exergy efficiency of China [31]–[38]. These results demonstrate the usefulness of exergy in solving environmental problems and progression toward the sustainable development of human society.

There are three main classifications for the exergy input and output with some sectors and society in these papers. Reistad's view: USA [39], OECD/World/non-OECD [40], Saudi Arabia [22]–[27]; Wall's view: China [16], [28], Norway [16], Italy [21], Japan [15]; Sciubba's view: Italy [41], Norway [42], China [37], [43].

Reistad's method only quantifies the exergy of energy carriers in an economy. Wall quantifies the exergy content of energy carriers and other materials such as metals, minerals, biomass as well as the waste emissions further. Sciubba adds capital and labor to the quantification of exergy using Wall's approach.

Their exergy analysis research results indicate the potential usefulness of exergy in addressing and solving environmental problems because exergy characterizes the largest amount of energy that can be extracted from material energy. Therefore, unlike energy flow, in which only the quantity is of concern, exergy is a measure of the quantity and quality of the energy and material resources.

Following Wall's approach, we construct an exergy flow network model of international ferrous metal ore trade to explore its features and patterns. In the international ferrous metal ore trade, there are not only flows of ores measured by tonnage and price but also flows of resource exergy, which forms an exergy flow network.

Complex network theory provides us with a good method to study this phenomenon. This theory considers the relationship among the variables in the real system as a complex network and allows understanding by analyzing the entire system [44]–[47]. This theory provides us with an appropriate method to resolve the action of networks in the economic system quantitatively, such as the small-world phenomenon [48]–[52]. Some researchers have studied the world trade web using the complex network [3], [53]–[57]. These researchers observed that the world trade web is a network and determined the scale-free features of the world trade web, which build upon the trade relationships between different countries in the world.

The countries are represented as nodes, and trade relations are represented as edges between these nodes. We omit the size and the wealth of the countries when analyzing the ferrous metal ore trade system at a global level. The objective of this paper is to determine the features of the exergy flow network of international ferrous metal ores from the view of exergy following Wall's concept and complex network theory. How does the role of a country change in the exergy flow network? Over time, what changes have occurred in the network? Does the exergy network exhibit the same features as the network of currency?

We organize our paper as follows: in the next section, we explain our method for transforming the trade network of ferrous metal ores into complex networks, and we report our results in the third section. The contrast between exergy and currency in average out strength and standard network strength entropy are considered in the fourth section. Finally, we summarize our work in the last section.

## Methodology and Data

### Data resources

This study collected data on iron ore (2601), chromium ore (2602) and manganese ore (261000) trade from *UN Comtrade*, which is a record of export and import flow in the world from 2002–2012. Our data were obtained from a publicly available database and can be downloaded from the web site http://comtrade.un.org/db/mr/daReportersResults.aspx; all the data underlying the findings described in this manuscript are freely available to other researchers.

### Calculating the exergy of the ferrous metal ores

When the environment is determined, the exergy of a system is determined as well. For the exergy of world ferrous metal ore accounts, it is reasonable to select a global standard environment to illustrate in the series of works on standard chemical exergy of some elements and compounds that facilitate further exergy calculations [58]–[60].

The chemical exergy contents of different material resources are represented in detail by Wall [7], [61], The exergy of substances and materials is given as



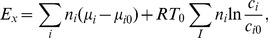
(1)where *T*_0_ is the temperature of the environment, *n_i_* is the *i*th mole number, 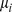 is the chemical potential of substance *i* in its present state, 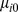 is the chemical potential of substance *i* in its environmental state, *c_i_* is the chemical concentration of substance *i* in its present state and *c_i_*_0_ is the chemical concentration of substance *i* in its environmental state.

Some researchers calculate the exergy of the metals and minerals in the standard environment [60], [62]–[64]. Chinese iron ore has an average iron content of approximately 35%; thus, the exergy content of the magnetite iron ore is calculated to be 0.42 MJ/kg. Iron concentrate ore has an iron content of approximately 70%, and the exergy is 0.84 MJ/kg. The exergy contents of manganese ore and chrome ore are estimated to be 0.2 MJ/kg and 0.4 MJ/kg using the same approach [14].

### Constructing the exergy flow network of global ferrous metal ores

In this study, we construct the directed complex network model of ferrous metal ores. The nodes are the countries, and the edges are the trade relationships of ferrous metal ores. The direction of the edges is the same as the direction of the ferrous metal ore trade. The exports represent the ferrous metal ores flowing out, and the imports represent the ferrous metal ores flowing in. To study the nature of the exergy flow of the global ferrous metal or complex network, we built an un-weighted directed complex network and a weighted directed complex network. In the un-weighted directed complex network, if country *i* exports ferrous metal ores to country *j* during year t, then a link from *i* to *j* is drawn, and 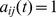. Otherwise, no link is drawn, and 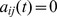.

In the weighted directed complex network, the properties of a graph can be expressed via its adjacency matrix 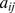, whose elements take the value 1 if an edge connects node *i* to node *j* and 0 otherwise (with *i, j*  =  1,....,N, where N is the size of the network). Weighted networks are usually described by a matrix 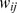, specifying the weight on the edge connecting the nodes *i* and *j* (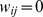 if the nodes *i* and *j* are not connected).

## Results and Analysis

### Node degree distribution and strength distribution

#### Node degree distribution

In the un-weighted complex network, the degree represents one node's connections with other nodes. The degree in the exergy flow network of global ferrous metal ore trade is the number of trade connections country i has with the other countries during year t. There are in-degrees and out-degrees in the directed network. The out-degree 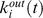 is the number of export links one country has with other countries, and the in-degree 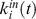 is the number of import links. The values of out-degrees and in-degrees can reflect the importance of one node in the network. These values are computed as



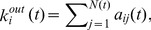
(2)

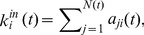
(3)where 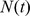 is the total number of nodes in the network. The out-degree of country 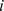 in year 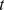 is the sum of 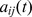, and the in-degree of country 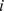 in year 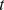 is the sum of 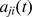. A node with a higher degree in the network will share more edges with other nodes. The situation is the same for import countries; however, we only study the situation for export countries in this paper.

**Figure 1:**
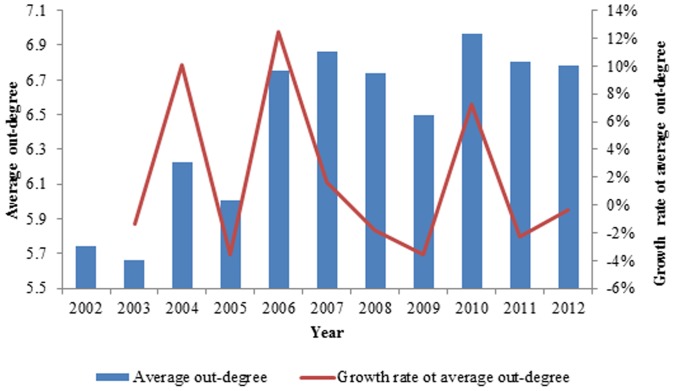
The evolution curve of the average out-degree from 2002 to 2012.

The average out-degree reflects the average connectivity of the network. Figure 1 presents the evolution curve of the average out-degree of the un-weighted directed network of the exergy flow of global ferrous metal ores. The abscissa is time, and the vertical axis is the average out-degree of ferrous metal ores.

**Figure 2:**
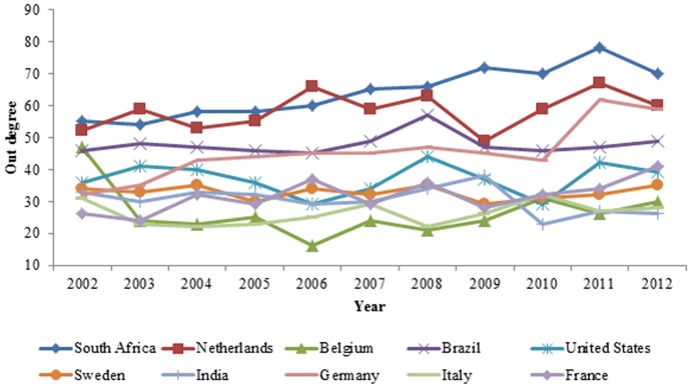
The change of the degree of the top 10 countries in 2002 from 2002 to 2012.

In Figure 1, the average out-degree increases with fluctuations from 5.739 in 2002 to 6.78 in 2012, which indicates that increasingly more countries have joined the international exergy network of ferrous metal ores and that the capacity of the network has expanded. Significant fluctuations of the growth rate occurred from 2003 to 2007. The growth rate of the average out-degree was −1.39% in 2003 and 10.04% in 2004, while more acute changes occurred in 2005 and 2006. Because of the great changes, the average out-degree increased from 5.739 in 2002 to 6.865 in 2007. Due to the financial crisis, some countries quit the network and caused the average out-degree to decrease in 2008 and 2009. Many countries joined in the network again, and the average out-degree rebounded to 6.966 in 2010, and the next fluctuation began. After that, these countries strengthened the relationships of ferrous metal ores in the world. The changes of the degrees of the top 10 countries from 2002 to 2012 are shown in Figure 2, and the ranks of the out degrees in the world from 2002 to 2012 are listed in Table 1.

**Table 1:** The rank of the out degree in the world from 2002 to 2012.

rank of out-degree	2002	2003	2004	2005	2006	2007	2008	2009	2010	2011	2012
South Africa	1	2	1	1	2	1	1	1	1	1	1
Netherlands	2	1	2	2	1	2	2	2	2	2	2
Belgium	3	10							7		10
Brazil	4	3	3	3	4	3	3	3	3	4	4
United States	5	4	5	5	8	5	5	6	10	5	6
Sweden	6	6	6	7	6	6	7	7	9	8	8
India	7	7	8	6	7	7	8	5			
Germany	8	5	4	4	3	4	4	4	4	3	3
Italy	9					8		9	5		
France	10	9	9	8	5	9	6	8	6	6	5
China		8	10							10	
Australia			7	9	9						
Turkey				10		10	9			7	7
Singapore					10						
Mexico							10				
Britain								10		9	9
Canada									8		

#### Node strength distribution

The topology description of the network is not clear when the intensity difference among the nodes has important effects on the network; for example, in the transportation network, transportation flow is an important parameter [65]. The weighted network provides a more accurate portrayal.

In the study of the exergy flow network of international ferrous metal ores, we use exergy flows to define the weights of the edges. “W” represents the adjacency matrix of the weighted network. If country *i* exports ferrous metal ores to country *j* in year t, then 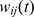 is the exergy flow of the ores.

Determining the most important node is the key issue of complex network research. The degree is the most direct measure of the importance of nodes in the topology network; nodes with higher degrees are more important. However, in the weighted network, some nodes with few connections but great flows will be ignored if we only consider their degrees. Therefore, the degree and exergy flow should both be considered in the weighted network.

In weighted networks, the concept of the node degree becomes node strength, representing the total exergy flow of ferrous metal ores of one country. Similar to the un-weighted network, the weighted networks have in strengths and out strengths. These values are computed using



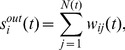
(4)

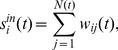
(5)where W_ij_(t) is the strength of node *i* to node *j* in year t, and N(t) is the number of countries in the international ferrous metal ore network in year t. The situation is the same for the in strength; however, we only study the circumstances of out strength in this paper.

**Figure 3:**
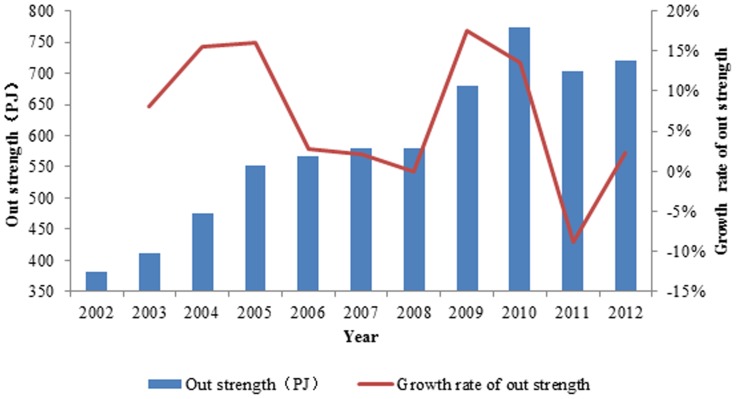
The evolution curve of the total out strength from 2002 to 2012.

The total out strength reflects the exergy flow of the ferrous metal ore exports of all the countries. The abscissa represents time, and the vertical axis represents the export exergy out strength of the ferrous metal ores. Figure 3 illustrates that the exergy flow of international ferrous metal ores grew rapidly from 2002 to 2005, which means that the export capability of the resource-exporting countries increased. The growth rate decreased over the following two years and became negative in 2008, indicating that the consequence of the financial crisis from the sub-prime crisis in the USA was so serious that it affected the exergy flow of international ferrous metal ores. The growth rate rebounded strongly in 2009 and 2010, revealing that the demand for ferrous metal ores underwent a worldwide boom after the crisis. The resource-exporting countries exported significantly more ferrous metal ores, which led the demand of resource-importing countries to decline; thus, the growth rate sharply declined in 2011 and rebounded again in 2012.

**Figure 4:**
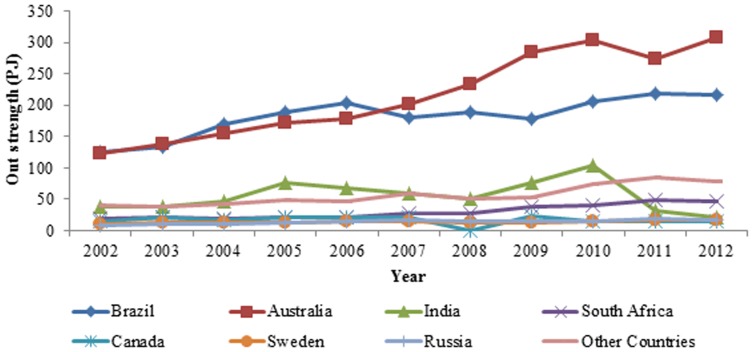
The evolution curve of out strength of the top 7 countries from 2002 to 2012.

**Figure 5:**
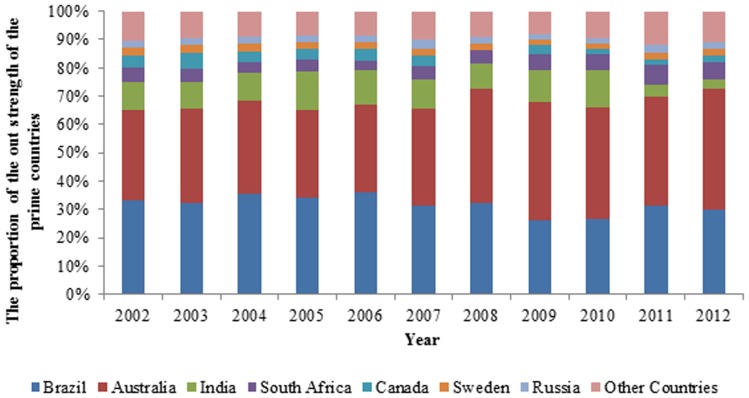
The out strength of the prime countries in the world from 2002 to 2012.

The evolution curves of the out strength of the top seven countries from 2002 to 2012 are presented in Figure 4 and Figure 5. These seven countries are Australia, Brazil, India, South Africa, Canada, Sweden and Russia. The abscissa is time, and the vertical axis is the exergy out strength of ferrous metal ores. The exergy out strength of the seven countries account for 90.37% of the world's total out-strength from 2002 to 2012, and that of other countries is less than 10%, which indicates that these seven countries are powerful and dominate the exporting ferrous metal ore network, as indicated in Table 2. Among these countries, the exergy out strengths of ferrous metal ores of Sweden, Russia and South Africa are growing; that of Canada is stable; that of India fluctuates greatly; and those of Brazil and Australia are in absolute dominance in the network, occupying more than 60% of the whole. In addition, their exergy out strengths increased significantly after the financial crisis. The ranks of the out strengths of the top countries in the world from 2002 to 2012 are listed in Table 3.

**Table 2:** The out strength of main countries from 2002 to 2012.

out strength (PJ)	2002	2003	2004	2005	2006	2007	2008	2009	2010	2011	2012
Brazil	126	133	170	188	203	180	188	178	206	219	216.5
Australia	122	137	155	171	178	201	233	284	303	273	306.6
India	38.4	38.2	46.9	76.1	66.9	58.5	50	76	104	30.7	22.1
South Africa	19	20.7	18.3	21.9	20.9	27	27.6	38	41.2	47.7	46.1
Canada	15.4	21.6	17.9	21.6	21.6	22	0.28	24.2	14.3	14.9	15.1
Sweden	10.7	11.7	13.1	13.3	13.9	14.4	13.3	12.1	15.6	15.9	17.2
Russia	8.76	11.1	11.4	12.2	15.2	17.1	15.1	13.8	14.7	18.2	17.2
Other Countries	40.74	38.7	43.4	47.9	47.5	59	51.72	53.9	74.2	84.6	79.2

**Table 3:** The rank of the out strength the top countries in the world from 2002 to 2012.

Rank of out strength	2002	2003	2004	2005	2006	2007	2008	2009	2010	2011	2012
Brail	1	2	1	1	1	2	2	2	2	2	2
Australia	2	1	2	2	2	1	1	1	1	1	1
India	3	3	3	3	3	3	3	3	3	4	4
South Africa	4	5	4	4	5	4	4	4	4	3	3
Canada	5	4	5	5	4	5		5	7	7	7
Sweden	6	6	6	6	7	7	6	7	5	6	5
Russia	7	7	7	7	6	6	5	6	6	5	6

**Figure 6:**
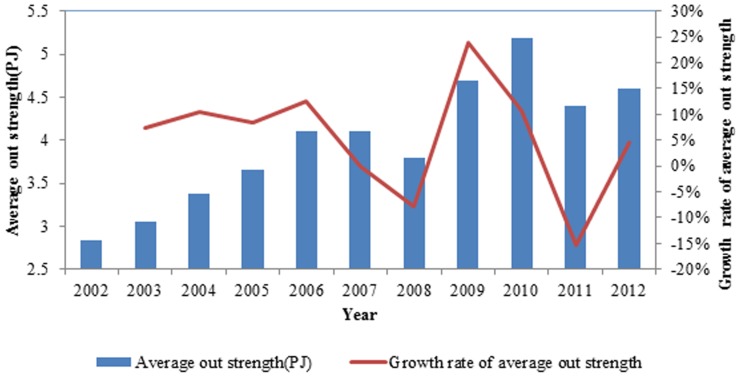
The evolution curve of the average out strength of the world from 2002 to 2012.

The average out strength reflects the average intensity of the international ferrous metal ore exports, which eliminates the effect of the number of countries on the out strength in Figure 6. The average out strength increased over the eleven years, while more countries with small exergy exports joined the network at the same time. Technological development resulted in a tremendous increase of international exergy flow in ferrous metal ores, promoting the increase of the average out strength.

**Figure 7:**
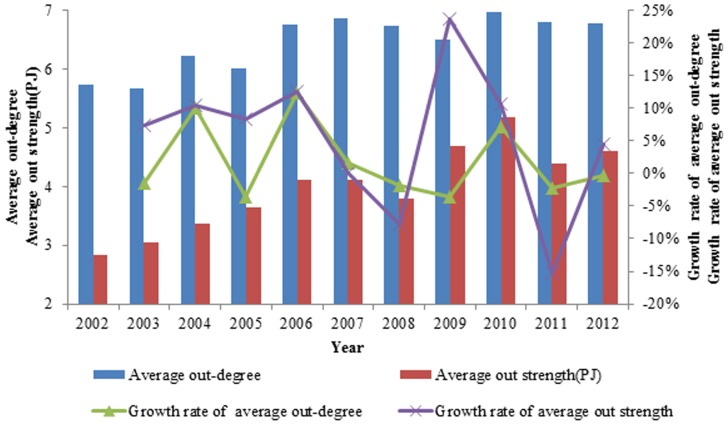
The contrast between the average out degree and the average out weighted.

The out strengths of a few nodes are so large that these nodes affect the average out strength of the entire network in Figure 7. The average out degree of the network fluctuates greatly, and the few nodes with large out strengths counteract this effect, causing the rate of change of the out strength to vary far less than the rate of change of the average out degree from 2003 to 2006. There is a large difference between the rate of change of the out strength and the rate of change of the out-degree. The former was 23.75% and the latter was −3.59% in 2009, while the former was lower than the latter 12.92% in 2011. In 2012, the former was 4.55% and the latter was −0.38%, still below zero. Therefore, in the exergy flow network of ferrous metal ores, a few nodes control most of the exergy current and affect the entire network.

### The standard network structure entropy and the standard network strength entropy

**Figure 8:**
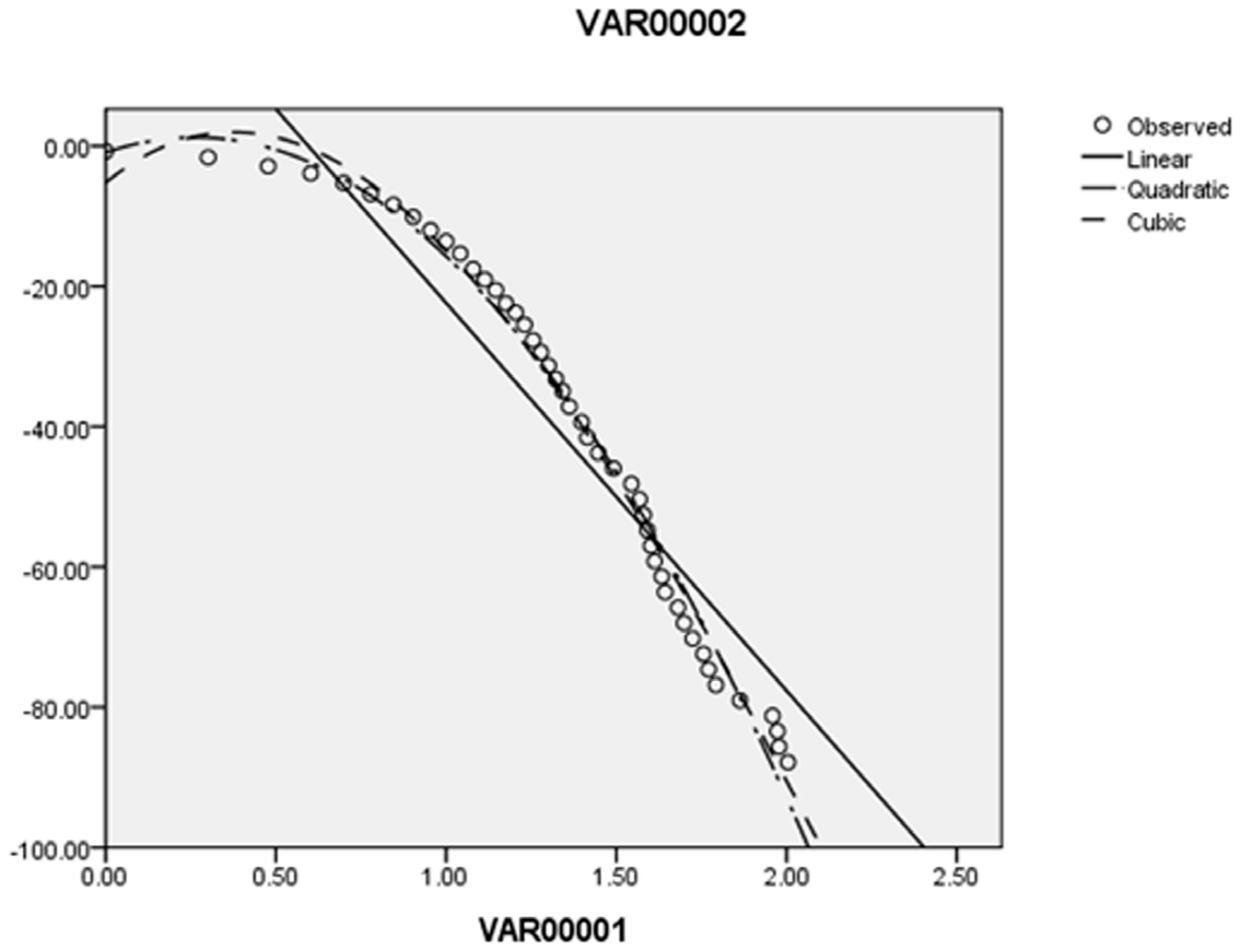
The curve of logarithmic cumulative degree distribution and logarithmic degree distribution.

The relationship between the cumulative degree distribution and degree distribution is linear in double logarithmic coordinates in scale-free networks. Figure 8 shows that the cumulative degree distribution and degree distribution are not linear in double logarithmic coordinates in 2011 (details please see the Table S1, Table S2 and Table S3), which is similar to the findings for the other years. Therefore, the exergy flow network of ferrous metal ores is not a scale-free network.

In the mode of Barabasi et al. [66], the new nodes in the networks are likely to connect with nodes with high degrees. Therefore, the central nodes are able to gain an advantage with their connections getting stronger. When the number of nodes grows to certain quantities, a few nodes with large connections emerge, and at the same time, many nodes have few connections.

In the exergy flow networks of international ferrous metal ores, new nodes are often countries with lower out strengths. Due to the difference of geography and transport cost, the preferential selection mechanism of scale-free networks cannot run fully in global exergy flow networks. While the preferential selection mechanism will work in part, the integrated exergy flow networks of international ferrous metal ores are not typical scale-free networks.

#### The standard network structure entropy

Essentially, scale-free complex networks are heterogeneous networks. A few core nodes in the network have numerous connections, and the majority of nodes have few connections [67]. We apply the network structure entropy and standard network structure entropy to study the network to describe the heterogeneity of the network of global ferrous metal ores [68]. The network structure entropy is given as



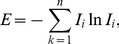
(6)where 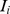 is the degree of importance of the ith node.

The degree of importance of the *i*th node is given as



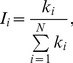
(7)where 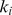 is the degree of *i*th node in the N nodes.

When the network is completely uniform, 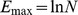, and when all nodes in the network are connected with a central node, 
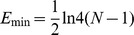
. To eliminate the effect of the number of nodes on the network structure entropy, we normalize the network structure entropy and obtain the standard network structure entropy, 
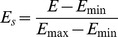
.

**Figure 9:**
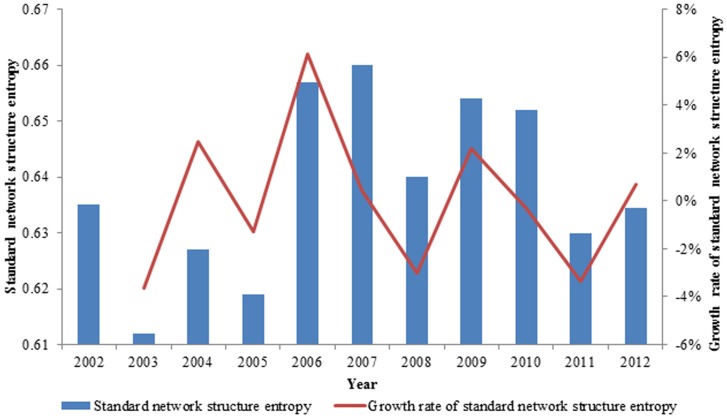
The evolution curve of the standard network structure entropy from 2002 to 2012.

We compare the heterogeneity of the exergy flow network of international ferrous metal ores in different years using the standard network structure entropy in Figure 9. The standard network structure entropy sharply decreased after a brief rise, which indicates that the heterogeneity of the network increased after a short-term fall. The network becomes mildly uniform and soon returns.

Over the past eleven years, the development of transportation and communication reduced the cost of the exergy flow of ferrous metal ores. However, ferrous metal ores are distributed unevenly around the world. The iron ore outputs in Brazil and Australia account for 37.6% of the world total, and these two countries account for 53.8% of the global reserve, calculating the iron content. The chrome ore in Kazakhstan and South Africa account for 87.5% of the world's reserves. Four countries, South Africa, Ukraine, Brazil and Australia, account for 78.3% of the world's manganese reserves [69]. With the financial crisis sweeping the globe, many countries quit the international ferrous metal ore network in 2008. A few countries did not leave the network because of their vast resources, which led to the fluctuation of the standard network structure entropy.

#### The standard network strength entropy

Similar to the network structure entropy and standard network structure entropy, we used the network strength entropy and standard network strength entropy [70] to describe the heterogeneity of the exergy flow weighted network of the international ferrous metal ores. The standard network strength entropy is given as
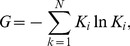
(8)where 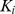 is the degree of importance of the *i*th node in the N nodes.

The degree of importance of the *i*th node is given as
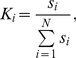
(9)where 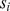 is the weighted strength of the *i*th node.

The network strength entropy indicates the uniformity of the weighted networks. When the network is completely uniform, that is, 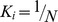, the network strength entropy is the maximum, 
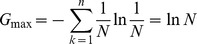
; and when all the weighted strength in the network concentrates at one node, that is, 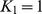, 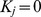 (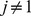), then, the network strength entropy is the minimum, 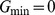.

To eliminate the effect of the number of the nodes on the network strength entropy, we normalized the network strength entropy and obtained the standard network strength entropy 
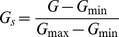
. The standard network strength entropy is able to describe the heterogeneity in the weighted networks.

**Figure 10:**
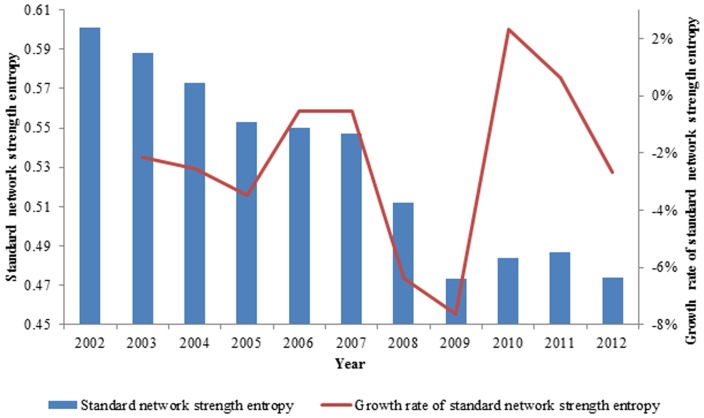
The evolution curve of the standard network strength entropy from 2002 to 2012.

After we calculated the standard network strength entropy of the exergy flow network of the ferrous metal ores, we observed that the gap of network strength is increasing, which indicates that the network is becoming increasingly heterogeneous and that the gap of the exergy out strength is growing in these countries, as demonstrated in Figure 10. It is similar to the social network of the rich-getting-richer phenomenon; more exergy flow is concentrated in major countries. The exergy flow network of international distribution tends to be more uneven, and the standard network strength entropy decreases from 0.601 to 0.474 from 2002 to 2012.

**Figure 11:**
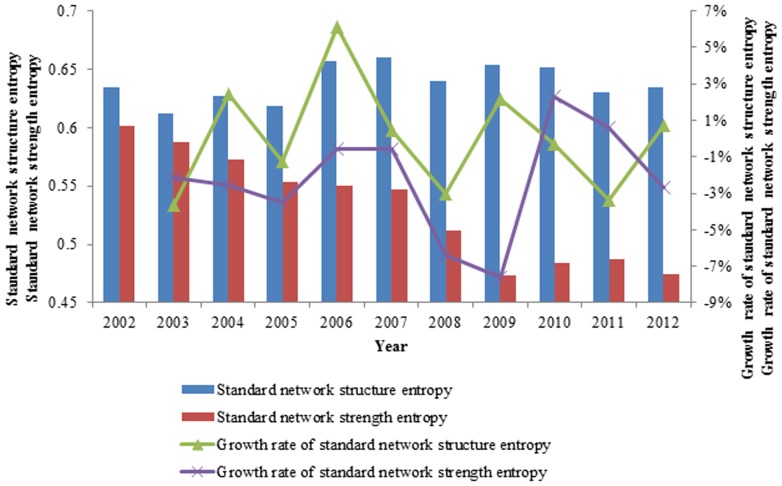
The contrast between the standard network structure entropy and the standard network strength entropy from 2002 to 2012.

In contrast to the standard network structure entropy, the standard network strength entropy containing the out strength of the nodes is a more realistic reflection of the exergy flow network of global ferrous metal ores. Compared with the standard network structure entropy, the value of the standard network strength entropy is lower, which means the unevenness of the entire network is more prominent, as shown in Figure 11. Overall, the standard network strength entropy decreases faster than the standard network structure entropy and decreases from 2002 to 2012 except in 2010 and 2011; the standard network structure entropy decreases with fluctuations. In short, although the new nodes have joined the network, the entire network is becoming increasingly controlled by a few nodes.

### Clustering coefficient and degree correlation coefficient

#### Clustering coefficient

The clustering coefficient describes the tightness between one node and its neighboring nodes. A node with more closely connected neighboring has a larger value of the clustering coefficient. We can determine the tightness of the exergy flow network of the ferrous metal ores by studying the clustering coefficient of the entire network [71]. The clustering coefficient is given as
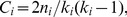
(10)where 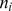 is the number of edges of the neighbors of node i in the un-weighted network with 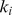 edges and *i* nodes.

When 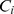 is zero, there is no link around node i; when 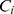 is 1, there are links among the neighbors of node i. A larger clustering coefficient indicates a better link among the neighbors of node i.

When we average the clustering coefficient of all the nodes in the network, we obtain the average clustering coefficient <C> [70]. The average clustering coefficient is given as
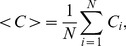
(11)where 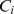 is clustering coefficient and N is the number of the nodes.

The average clustering coefficient describes the average clustering degree of the entire network. The trend of the average clustering coefficient is growing, which indicates that with the development of the process of globalization, regional exchanges are becoming important, and regional grouping appears in the exergy flow network of international ferrous ores.

**Figure 12:**
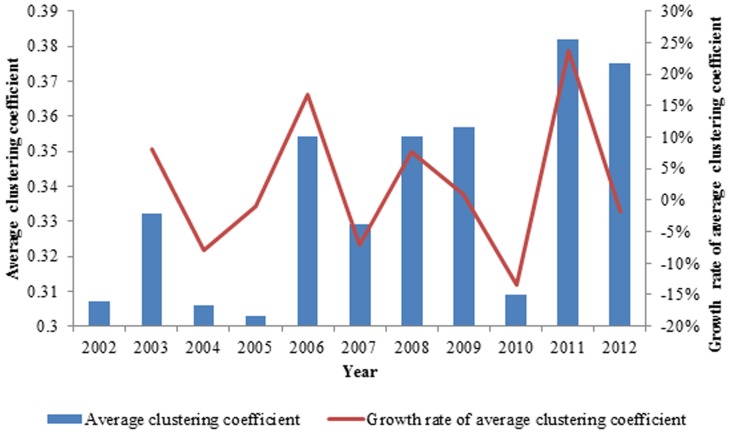
The evolution curve of the average clustering coefficient from 2002 to 2012.

The average clustering coefficient of the exergy network of the ferrous metal ores increased from 0.307 in 2002 to 0.375 in 2012 according to Figure 12. The overall average clustering coefficient fluctuates acutely each year. The average clustering coefficient is high one year and declines greatly in the next year and exhibits an overall upward trend over the eleven years.

#### Degree correlation coefficient

Many real world networks contain different types of nodes, and the probability of the links among the node often relies on the types of nodes. When one node with a high degree is prone to connect with other high-degree nodes, the result is called a similar mix; when one node with a high degree is prone to connect with low degree nodes, the result is called a non-similar mix. Vazquez [72] proposed one method to calculate the degree correlation coefficient,



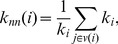
(12)where 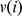 represent the nodes close to the node i. We averaged the degree of the nodes of K nearest neighbors [72]:



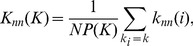
(13)


If 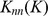 increases with the increase of K, and, at the same time, the node exhibits a trend to link the same or greater degree nodes, the network is called a similar mixed network. In contrast, if 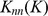 decreases with the increase of K, and, at the same time, the low degree nodes exhibit a trend to link the pivot nodes, the network is called a non-similar mixed network. Newman [73] simplified the calculation method using



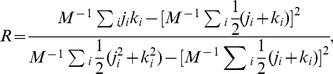
(14)where 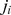 and 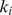 represent the degree of the i-th node of the edge ends and M represents the number of edges.

When the network is a similar mixed network, the R-value is positive, and when the network is a non-similar mixed network, the R-value is negative. An R-value less than zero indicates that the network is a non-similar hybrid network. Countries with little degrees tend to connect with countries with high degrees, which indicates that due to geographical proximity and similar culture, small countries tend to develop relations with the hub country of the region; thus, a regional cooperation organization with regional power as its core is formed. Regional cooperation organizations develop continually based on the cooperation among member states, and the growth of the regional organizations make all the member states receive benefits from the expansion of regional markets. This result leads to the reinforcement of complementary advantages among member countries and the efficient allocation of resources in the region.

**Figure 13:**
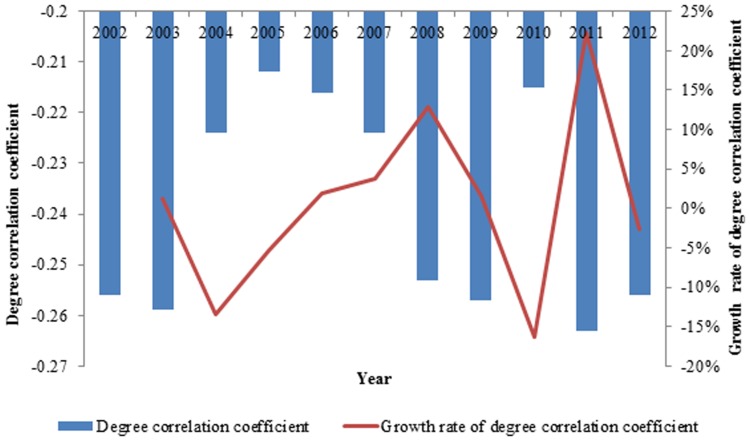
The evolution curve of the degree correlation coefficient from 2002 to 2012.

In the established regional organization, regardless of their level of integration or the type of cooperation, internal partnerships among these countries are always closer than their external relations, and the level of mutual opening among these countries is higher. However, ferrous metal ore globalization requires regional organizations connected with others, not forming one closed regional group. In fact, these organizations maintain and develop relationships with other regions and countries and are not completely isolated. The dominant country plays a pivotal role in the region, becoming a bridge to the rest of the world. Therefore, the formation of the international ferrous metal ore system globalization and regional integration coexist. The increase of the average clustering coefficient <C> indicates that this trend is improving. We can determine the variation of the degree correlation coefficient R from Figure 13.

The correlation coefficient of the network has a close relationship with the network survivability. High degree nodes cluster locally in a similar mixed network. The entire network remains well connected, and when these nodes are removed, not much effect will be observed on the network. However, in a non-similar mixed network, when a few high degree nodes are removed, the entire network is devastated. For the high degree nodes to distribute widely in the network, these nodes must link to many other nodes and provide are important support to the network connectivity.

In the networks of the exergy flow of ferrous metal ores, the value of R is declining, meaning that the nodes with more relationships are distributed more extensively. The entire networks may be seriously damaged if these nodes with more relationships are removed.

### The contrast between exergy and currency in the average out strength and standard network strength entropy

#### The contrast of the average out strength of exergy and the average out strength of currency

**Figure 14:**
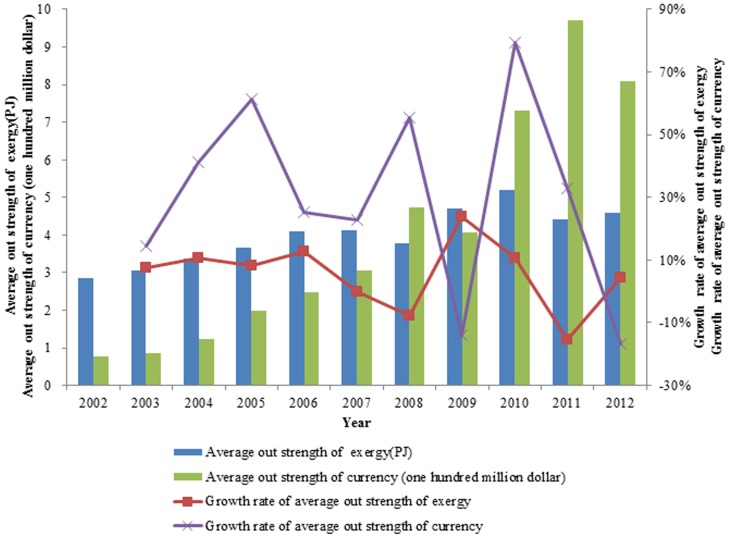
The contrast of the average out strength of exergy and the average out strength of currency from 2002 to 2012.

Figure 14 demonstrates that the average out strength of the international ferrous metal ores network grew from 2002 to 2007 both in terms of exergy and currency. When the effect of the financial crisis reached ferrous metal ores, the world demand for ferrous metal ores decreased; thus, the average intensity of the exergy flow network decreased in 2008. The booming economies of emerging countries such as China have invested numerous resources in infrastructure, which has led the demand for ferrous metals to increase rather than decrease. Resource-export countries such as Brazil and Australia exporting a large proportion of the ferrous metal ores in the world have raised the prices of the ferrous metal ores artificially, which caused the price to sky-rocket and the average out strength of currency to increase as well.

After the financial crisis, the global economy began to recover, and the demand for ferrous metal ores increased; therefore, the average out strength of exergy recovered in 2009 and 2010. The law of value causing the price of the ferrous metal ores to be lower than in 2008 resulted in the decrease of the average out strength of currency in 2009. Likewise, the average out strength of currency increased in 2010. The continuous growth of the average out strength of exergy in 2009 and 2010 decreased the global demand, which caused the average out strength of exergy to decrease in 2011 and rebound slightly in 2012. Some resource–export countries monopolizing the exporting market raised the price of ferrous metal ores to protect their own interests. When the financial crisis began, the quantitative easing of the United States caused the international commodity prices sky-rocket and increased the average out strength of the currency.

Prices are affected by human perception factors to a large extent; thus, the growth rate of the average out strength of currency fluctuated acutely in the ten years from 2002 to 2012. Exergy is defined as the maximum work that can be extracted from a system and can reflect the true global cost for a commodity.

#### Contrast of the standard network strength entropy of exergy and the standard network strength entropy of currency

**Figure 15:**
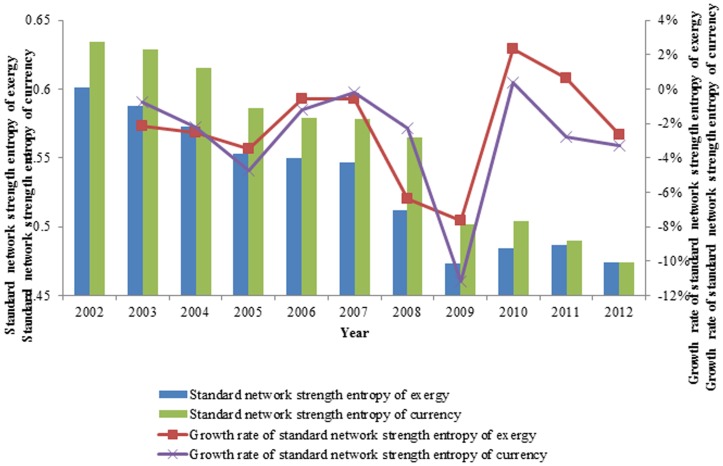
The contrast of the standard network strength entropy of exergy and the standard network strength entropy of currency from 2002 to 2012.

Analyzing the two aspects of exergy and currency, we observe that the changes of the weighted entropies of the standard networks are consistent. A larger value of standard network weighted entropy indicates a smaller heterogeneity of the network. That is, the network is more uniform. Conversely, a smaller value of the standard network weighted entropy indicates a greater heterogeneity of the network, and the network is not uniform. In Figure 15, the trend of the value of the standard network weighted entropy is decreasing, indicating that the network is becoming increasingly uneven. In other words, the out weighted degree of the networks is becoming increasingly controlled by a few countries.

In 2009, when the world economy just recovered from the financial crisis, the standard network weighted entropy was at its minimum value, and most of the out strength was controlled by a few countries. In 2010, the world demand increased, and some other countries joined the network, which reduced the proportion of the main countries. Therefore, the value of the standard network weighted entropy increased slightly. In 2011, the demand for ferrous metal ores decreased, and some countries quit the network, which increased the proportion of the main countries; therefore, the value of the standard network weighted entropy decreased slightly, as occurred again in 2012.

## Concluding Remarks

In this paper, we have explored the exergy flow network of the world's ferrous metal ores using complex network analysis.

The average out degree of the ferrous metal ores exergy network increases with fluctuations from 2002 to 2012, indicating that more countries joined the network. The out strength not only reflects the degree of one node but also reflects the exergy flow of the node, which is more similar to reality. For example, the degrees of Australia and Brazil are not high; however, the out strengths of the two countries are high, and the sum of the two countries accounts for 67.33% of the world's total exergy flow. In other words, a few nodes dominate most of the exergy flow in the ferrous metal ore network. A similar result was obtained in [3], [4]. While some new nodes have joined the networks of the exergy flow of ferrous metal ores during these ten years, the entire network is uneven.

The trend of the average clustering coefficient is growing in the ferrous metal ores network, which means that with the development of globalization, regional exchanges are becoming important, and regional grouping appears among the countries.

Countries with low degrees tend to connect with countries with high degrees; thus, there are regional cooperation organizations with regional power. Regional cooperation organizations develop continually based on the cooperation among their members. Therefore, global and regional ferrous metal ore systems coexist.

**Figure 16:**
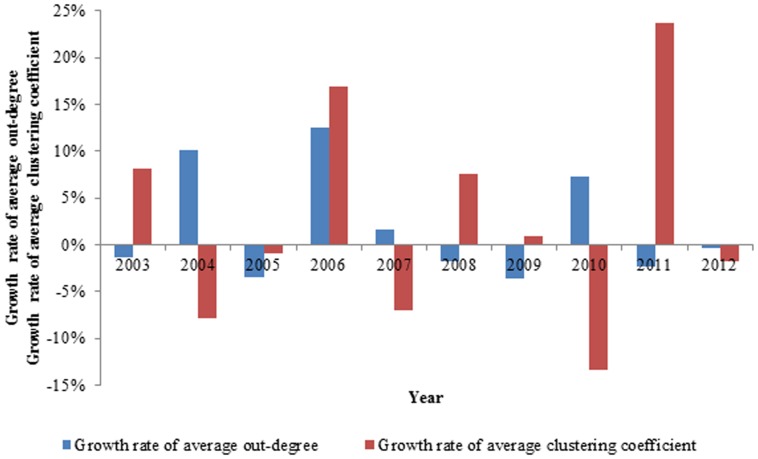
The contrast between the growth rate of average clustering coefficient and the growth rate of average out-degree.

We observed that the growth rate of the average clustering coefficient and the growth rate of the average out-degree changed in opposite directions except in 2005, 2006 and 2012, as demonstrated in Figure 16. In most cases, when more countries are in the network, the relationships among countries are relatively loose, and vice versa. While the generality of this concept may be expected, our approach quantifies this phenomenon in detail.

The average out strengths of exergy and currency have similarities and differences. The growth rate of the average out strength of currency fluctuates acutely over the ten years because the prices involve too much human perceptions. Exergy, defined as the maximum work that can be extracted from a system, can reflect the true cost and fluctuates much less. Performing an analysis from the aspects of exergy and currency, we observed that the changes of the weighted entropies of the standard networks are consistent: the network is becoming increasingly uneven.

## Supporting Information

Table S1 **Test results: Model Summary.**
(PDF)

Table S2 **Test results: Anova.**
(PDF)

Table S3 **Test results: Coefficients.**
(PDF)
