# Supplementary material for: Analyzing the International Exergy Flow Network of Ferrous Metal Ores
Source: PLoS One. 2014 Sep 4;9(9):e106617. doi: 10.1371/journal.pone.0106617 (PMC4154736; doi:10.1371/journal.pone.0106617)
Supplement: Table S1 — Test results: Model Summary. (PDF) [file pone.0106617.s001.pdf]

Table S1 Test results: Model Summary

	R	R Square	Adjusted R Square	Std. Error of the Estimate
**Linear**	0.941	0.886	0.883	9.137
**Quadratic**	0.993	0.987	0.986	3.171
**Cubic**	0.994	0.989	0.988	2.937

The independent variable is VAR00001
