# Supplementary material for: Analyzing the International Exergy Flow Network of Ferrous Metal Ores
Source: PLoS One. 2014 Sep 4;9(9):e106617. doi: 10.1371/journal.pone.0106617 (PMC4154736; doi:10.1371/journal.pone.0106617)
Supplement: Table S2 — Test results: Anova. (PDF) [file pone.0106617.s002.pdf]

Table S2 Test results: ANOVA

		Sum of Squares	df	Mean Square	F	Sig.
**Linear**	Regression	28539.695	1	28539.695	341.870	.000
	Residual	3673.175	44	83.481		
	Total	32212.870	45			
**Quadratic**	Regression	31780.487	2	15890.243	1580.265	.000
	Residual	432.384	43	10.055		
	Total	32212.870	45			
**Cubic**	Regression	31850.597	3	10616.866	1230.863	.000
	Residual	362.273	42	8.626		
	Total	32212.870	45			

The independent variable is VAR00001.
