# Supplementary material for: Analyzing the International Exergy Flow Network of Ferrous Metal Ores
Source: PLoS One. 2014 Sep 4;9(9):e106617. doi: 10.1371/journal.pone.0106617 (PMC4154736; doi:10.1371/journal.pone.0106617)
Supplement: Table S3 — Test results: Coefficients. (PDF) [file pone.0106617.s003.pdf]

Table S3 Test results: Coefficients

		Unstandardized Coefficients		Standardized Coefficients		
		B	Std. Error	Beta	t	Sig.
**Linear**	VAR00001	-55.269	2.989	-.941	-18.490	.000
	(Constant)	32.886	4.195		7.839	.000
**Quadratic**	VAR00001	16.343	4.122	.278	3.965	.000
	VAR00001 ** 2	-31.155	1.735	-1.260	-17.953	.000
	(Constant)	-.929	2.381		-.390	.698
**Cubic**	VAR00001	39.639	9.019	.675	4.395	.000
	VAR00001 ** 2	-57.496	9.378	-2.326	-6.131	.000
	VAR00001 ** 3	8.177	2.868	.690	2.851	.007
	(Constant)	-5.234	2.672		-1.959	.057

The independent variable is VAR00001
